# Urban Chikungunya in the Middle East and North Africa: A systematic review

**DOI:** 10.1371/journal.pntd.0005707

**Published:** 2017-06-26

**Authors:** John M. Humphrey, Natalie B. Cleton, Chantal B. E. M. Reusken, Marshall J. Glesby, Marion P. G. Koopmans, Laith J. Abu-Raddad

**Affiliations:** 1 Division of Infectious Diseases, Department of Medicine, Weill Cornell Medicine, New York, New York, United States of America; 2 Viroscience department, Erasmus University Medical Centre, Rotterdam, The Netherlands; 3 National Institute for Public Health and the Environment (RIVM), Bilthoven, The Netherlands; 4 Department of Healthcare Policy and Research, Weill Cornell Medicine, Cornell University, New York, New York, United States of America; 5 Infectious Disease Epidemiology Group, Weill Cornell Medicine in Qatar, Cornell University, Qatar Foundation, Education City, Doha, Qatar; 6 College of Public Health, Hamad bin Khalifa University, Qatar Foundation, Education City, Doha, Qatar; Centers for Disease Control and Prevention, UNITED STATES

## Abstract

**Background:**

The epidemiology of *Chikungunya virus* (CHIKV) in the Middle East and North Africa (MENA) is not well characterized despite increasing recognition of its expanding infection and disease burden in recent years.

**Methodology / Principal findings:**

Following Cochrane Collaboration guidelines and reporting our findings following PRISMA guidelines, we systematically reviewed records describing the human prevalence and incidence, CHIKV prevalence/infection rates in vectors, outbreaks, and reported cases for CHIKV across the MENA region. We identified 29 human seroprevalence measures, one human incidence study, one study reporting CHIKV infection rates in *Aedes*, and nine outbreaks and case reports/series reported in the MENA from 1970–2015. Overall, anti-CHIKV antibody or reports of autochthonous transmission were identified from 10 of 23 countries in the MENA region (Djibouti, Egypt, Iraq, Iran, Kuwait, Pakistan, Saudi Arabia, Somalia, Sudan, and Yemen), with seroprevalence measures among general populations (median 1.0%, range 0–43%) and acute febrile illness populations (median 9.8%, range 0–30%). Sudan reported the highest number of studies (n = 11) and the highest seroprevalence among general populations (median 12%, range 0–43%) and undifferentiated acute febrile illness populations (median 18%, range 10–23%). CHIKV outbreaks were reported from Djibouti, Pakistan, Sudan, and Yemen.

**Conclusions / Significance:**

Seroprevalence studies and outbreak reports suggest endemic transmission of urban cycle CHIKV in at least the Red Sea region and Pakistan. However, indications of seroprevalence despite a low quantity of CHIKV epidemiologic research from the region suggests that CHIKV transmission is currently underrecognized.

## Author summary

*Chikungunya virus* (CHIKV) is an alphavirus whose principal vectors are the *Aedes aegypti* and *Aedes albopictus* mosquitoes. Though long endemic to Asia and Africa, detection of CHIKV has recently been reported throughout the Western Hemisphere, including much of South America and the Caribbean. In the Middle East and North Africa (MENA), the epidemiology of CHIKV remains poorly characterized despite recent reports of outbreaks and novel transmission in the Arabian Peninsula. To better understand existing data describing the epidemiology of urban CHIKV in the MENA region, we conducted a systematic review of human prevalence studies and incidence studies; CHIKV detections, prevalence, and infection rates in *Aedes*; and reported CHIKV outbreaks, case series, and case reports from the region. A total of 29 seroprevalence studies were identified through our search, with anti-CHIKV antibodies and/or outbreaks detected in Djibouti, Egypt, Iraq, Iran, Kuwait, Pakistan, Saudi Arabia, Somalia, Sudan, and Yemen. Sudan reported the highest number of studies (n = 11) and the highest seroprevalence among all studies. The epidemiology of urban CHIKV in other MENA countries is less well characterized, suggesting underascertainment of cases and the need for further research.

## Introduction

*Chikungunya virus* (CHIKV) is an alphavirus whose recognized global distribution increasingly overlaps that of dengue and the distribution of their shared mosquito vectors, *Aedes aegypti* and *Aedes albopictus* [1]. Clinical reports suggest that CHIKV may have been broadly distributed by the 1800s, though its similar clinical presentation to *Dengue virus* (DENV) makes its historic epidemiology uncertain [2]. Since the first isolation of CHIKV in Tanzania in 1952–53 [3], large urban outbreaks have been detected in the Indian Ocean region and Latin America, yielding millions of suspected infections and the novel discovery of autochthonous CHIKV transmission in Mediterranean Europe in 2007 [4–9]. The past decade has witnessed novel reports of CHIKV outbreaks in the Middle East and North Africa (MENA) as well. However, the present and historic epidemiology of CHIKV in this region remains poorly characterized.

Over the years, limited surveillance and diagnostic capacity have likely hindered the recognition of CHIKV in the MENA region. Although the earliest possible clinical description of CHIKV infection known to exist was first recorded in Cairo, Egypt in 1658 [2], it was not until 2011 that the presence of CHIKV was first confirmed (i.e. by viral culture or molecular detection) in the MENA during an outbreak in Yemen with over 15,000 suspected cases [10]. The CHIKV lineage responsible for this epidemic was related to the enzoonotic East-Central-South African (ECSA) Indian Ocean Lineage and was isolated from *A*. *aegypti*, a finding that suggested urban cycle transmission [11, 12]. To date, the existence of a sylvatic transmission cycle has not been reported in the MENA region. Although debilitating and prolonged polyarthralgias can be a recognizable hallmark of CHIKV infection [1], the clinical syndrome can be difficult to distinguish from other mosquito-transmitted febrile illnesses including dengue fever [2, 10, 13], o’nyong’nyong fever [2], yellow fever [14], and malaria [14]. Given the increasing global impact of *Aedes*-transmitted arboviruses and the limited knowledge of urban CHIKV in the MENA region, we performed a systematic review of the literature to describe the published evidence pertaining to the epidemiology of CHIKV in the MENA region.

### Objectives

The objective of this study was to characterize the epidemiology of urban CHIKV in the MENA region through a systematic review of published human prevalence and incidence studies, human outbreaks and reported cases, and studies reporting CHIKV detections and prevalence/infection rates in *Aedes* mosquitoes. The original literature search was conducted in December 2015 and updated in May 2017.

## Materials and methods

The materials and methods used for this review are similar to those of a systematic review of DENV in the MENA region that we conducted in parallel to the current study [15].

### Eligibility criteria

The eligibility criteria for this study follows similar criteria that was used for a review of DENV in the MENA region (Table 1) [15]. In brief, reports containing primary human seroprevalence or incidence, outbreaks and reported cases, and CHIKV detections from *Aedes* mosquitoes in the MENA region published in any year were considered eligible for the systematic review. For incidence studies, those that reported the number of acute infections or seroconversions over any time interval, or overall attack rate if assessed during an outbreak, were eligible. Human CHIKV outbreaks, case series, and case reports in natives and returned travelers from the MENA region were also sought from the articles retrieved through the search databases using the original search criteria. Outbreak reports were included if at least some of the reported cases were laboratory-supported CHIKV infection; cases series and case reports were only included if they were laboratory-supported. We considered any report of CHIKV cases to constitute an outbreak if the author of the report qualified it as such. As with DENV, there is currently no consensus on how to define CHIKV outbreaks, so determining whether any number of cases represents a significant deviation from baseline transmission is often unclear [15].

**Table 1:** Criteria for study inclusion or exclusion.

Study type	Inclusion Criteria	Exclusion Criteria
Human prevalence and incidence		
publication characteristics	Full article or abstract published in any year, language, setting, or human population in the MENA region; any seroconversion interval for incidence studies or population-based attack rate	editorials, letters to editors, reviews, commentaries, qualitative studies, basic science research studies, studies conducted in countries outside the MENA region, studies conducted in animals
study design	Any randomized or non-randomized design	Non-empirical research/modelled data
outcomes	CHIKV seroprevalence or prevalence of laboratory-confirmed infection; CHIKV incidence (by any laboratory method)	No human prevalence or incidence measure reported
Human outbreaks	Any outbreak defined as such in the report; reports may include laboratory-confirmed and suspected cases	No laboratory-supported information that CHIKV was the pathogen
Human case reports and case series	Any cases reported in MENA natives or in returned travelers from a MENA country, confirmed by any laboratory method	No laboratory method reported
Virus prevalence in vectors	Reported CHIKV prevalence in *Ae*. *aegypti* or *Ae*. *albopictus* pools (i.e. absolute number of positive pools); estimated infection rates (minimum/maximum) by any laboratory method	Basic science research studies, virus prevalence or CHIKV detections in other mosquito species or in non-MENA country
Single virus isolations in vectors	Any reports of single CHIKV isolates or vRNA detections from *Ae*. *aegypti* or *Ae*. *albopictus* obtained by any laboratory method	Mosquito captured in non-MENA country

Finally, studies containing CHIKV prevalence in *Aedes* pools and single CHIKV isolates or vRNA detections from *Aedes* were included if they contained a measure of the estimated proportion of CHIKV-infected *Ae*. *aegypti* or *Ae*. *albopictus* at a given time and setting in the MENA region. Prevalence studies in animals were noted but excluded from the systematic review, as our study focused on urban cycle CHIKV mediated by human-mosquito transmission. Our review covered the 23 countries included in the MENA definitions of the WHO/EMRO, World Bank, and the Joint United Nations Programme on HIV/AIDS (UNAIDS) for consistency with our systematic review of DENV in the MENA region as well as our earlier regional analyses of various infectious diseases such as HIV and other sexually transmitted infections and *Hepatitis C virus* [16–19].

### Outcomes

For the systematic review, the primary outcomes were CHIKV human seroprevalence, human CHIKV incidence, human case reports/case series of CHIKV infection in MENA natives and returned travelers from the MENA region, CHIKV prevalence in *Aedes*, and single virus isolates of CHIKV in *Aedes* in the MENA region.

### Data sources and search strategy

We conducted a systematic search for CHIKV in the MENA informed by the Cochrane Collaboration guidelines [20] and reported our findings using the Preferred Reporting Items for Systematic Reviews and Meta-analyses (PRISMA) guidelines [21]. The PRISMA checklist is found in S1 Table and our search criteria in S1 Fig. We searched PubMed (indexed since 1966 and selectively since 1865) and Embase (indexed since 1988) using text and MeSH/Emtree terms exploded to include all subheadings, as well as the World Health Organization (WHO) Index Medicus for the Eastern Mediterranean Region, WHO African Index Medicus (both indexed since 1984), and ProMED-MENA (indexed since 1994) using only the search term “chikungunya”.

### Study selection

The methodology for this section also follows our previous review of DENV [15]. Titles and abstracts were imported into Endnote (Thompson Reuters, Philadelphia, PA, USA) and were screened by one author (JH) with potential eligibility determined by consensus with a second author (NC) when eligibility was unclear. Full texts of potentially relevant records were retrieved and assessed for eligibility, contacting the author of the report as necessary. Reference lists of all potentially eligible articles and reviews were also searched. For this study, ‘report’ refers to the document (paper, abstract, or public health record) containing an outcome measure of interest, while ‘study’ refers to the outcome measure(s) within that report. Hence, reports could contribute more than one study, though multiple reports of the same study were counted only once.

### Data extraction and synthesis

Data were extracted by one of the authors (JH). Data from reports in English were extracted from the full texts, while reports in French (n = 1) and German (n = 1) were extracted from the English abstract and with the help of online language software and French and German language speakers [22]. There were no records in other languages. Studies were compiled by country and organized by year. Prevalence studies were stratified as follows: 1) *general prevalence studies* assessing anti-CHIKV IgG prevalence (e.g. CHIKV exposure) among individuals not suspected to have acute CHIKV infection, including community members, blood donors, military, students, and hospitalized patients and outpatients receiving care for non-febrile illnesses, and 2) *acute febrile illness studies* assessing the prevalence of laboratory-supported CHIKV infection (i.e. any positive laboratory test suggesting CHIKV infection) in those with undifferentiated acute febrile illness (AFI) or suspected arbovirus infection (IgG prevalence measures obtained during the acute phase of illness in these studies are presumed to reflect an earlier infection). These stratifications were made because of the different study aims and pretest probabilities of having laboratory evidence of CHIKV infection in these populations. Reports were summarized by country and year, along with the sampling method, assay type/make, sample size, and any data pertaining to alphavirus cross-reactions that was available.

For CHIKV outbreaks, we recorded the year, location, suspected or confirmed vector, and number of cases as provided in each report. We recorded similar information for case reports/series and cases in returned travelelers as available. Finally, reports containing CHIKV prevalence, infection rates, and single virus isolations in *Ae*. *aegypti* and *Ae*. *albopictus*, both recognized urban cycle CHIKV vectors, were also sought using the original search criteria. We recorded the year, location, and methods of collection and laboratory methods, as well as additional bioecological aspects of the vectors as available. The geographic distribution of all included human prevalence studies, outbreak reports, and case reports/series were mapped according to the first-level administrative division (e.g. state, province) in which each event was recorded (Tableau Software, Seattle, WA, USA).

### Risk of bias assessment

The risk of bias (ROB) was assessed for each seroprevalence study based on the Cochrane approach [20] and by evaluating the precision of the reported measures according to previously developed methodology [23, 24]. Each CHIKV seroprevalence measure was considered to have a low, high, or unclear ROB in two domains: sampling methodology and response rate. The latter was defined as the number of tested individuals divided by the number of persons invited to participate in the study [25]. ROB was considered low if (1) sampling was probability-based (using some form of random selection), and (2) response rate was ≥80%. Studies with missing information for either of the domains were classified as having unclear ROB for that specific domain. We did not assess the ROB for the sampling methodology of populations with acute febrile illness, as these are defined populations presenting to a health facility with acute infection and no population-based sampling is needed to capture these populations. We also did not include the laboratory assay characteristics in our risk of bias assessment given that the epidemiology of antigenically similar alphaviruses (e.g. *O’nyong-nyong virus* [ONNV], *Sindbis virus* [SINV], and *Semliki Forest virus* [SFV]) in the MENA was also unclear, and nearly all identified studies utilized in-house assays. Studies were considered to have high precision if the number of individuals tested was ≥ 100. We considered this to be a reasonably sensitive cutoff for precision given the heterogeneous epidemiology of CHIKV across the region (e.g. a prevalence of 1% has a 95% CI of 0–3%).

## Results

### Search results

**Fig 1:**
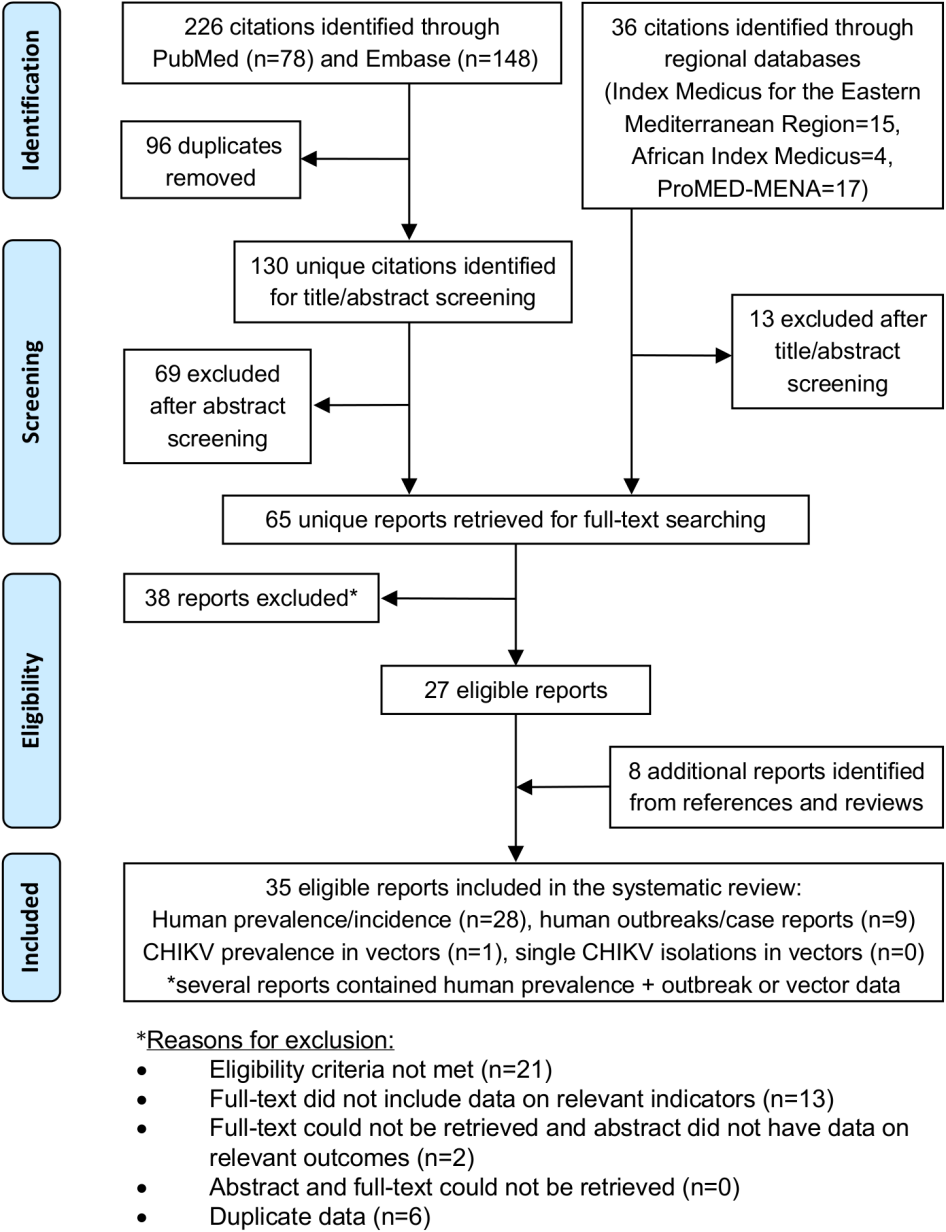
PRISMA flow diagram of report selection in the systematic search.

The selection process based on PRISMA guidelines is illustrated in Fig 1 [21]. Briefly, the search yielded 262 reports, 35 of which were eligible for inclusion in the study following screening process and after the addition of 8 reports identified from reference lists and reviews. One animal seroprevalence study was excluded, in which anti-CHIKV antibody prevalence of 2.5% was reported in a sample of 157 rodents captured in Pakistan. In this study, all antibodies were cross-reactive with SINV [26].

### Characteristics of included studies

**Fig 2:**
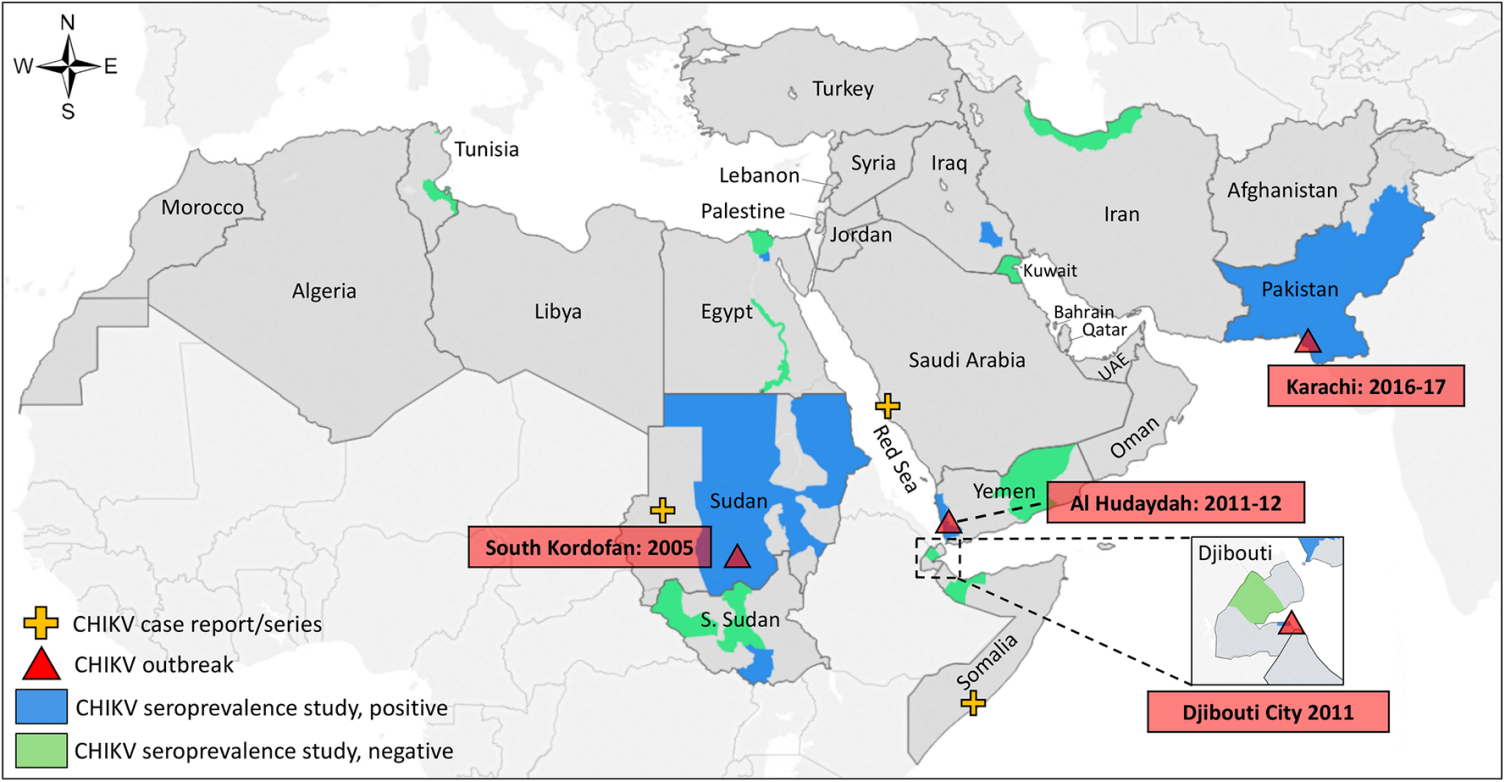
Geographic distribution of human prevalence studies and reported outbreaks and cases for *Chikungunya virus* in the Middle East and North Africa.

A total of 29 human seroprevalence studies for CHIKV were identified from eligible reports, 62% of which were conducted prior to 1990 (Table 2). Fig 2 illustrates the geographic distribution of all published CHIKV prevalence studies, outbreaks, and reported cases in the MENA region. Overall, anti-CHIKV antibodies were reported from 10 of 23 countries in the MENA: Djibouti, Egypt, Iran, Iraq, Kuwait, Pakistan, Saudi Arabia, Somalia, Sudan, and Yemen. The median seroprevalence measures among general populations was 1.0% (range 0–43%), and 9.8% (range 0–30%) among populations with acute febrile illness. Sudan reported both the highest number of studies (n = 11) and the highest seroprevalence measures overall (median 12.9%, range 0–43%). Ninety-three percent of all studies utilized in-house assays; 82% of all studies that were conducted after 1990 (n = 11) utilized ELISA and/or RT-PCR (or RT-qPCR); 56% (10/18) of studies prior to 1990 utilized hemagglutinin inhibition (HI) assays. Serologic cross-reactions with related alphaviruses (e.g. ONNV, SINV, and SFV) or travel-acquired infections were observed or could not be excluded in some studies from Djibouti [27], Iran [28], Kuwait [29, 30], and Pakistan [26]. Viral neutralization testing was performed in a total of five studies from Djibouti [31], Iraq [32, 33], and Sudan [32–34]. Among acute febrile illness studies, the prevalence of laboratory-supported CHIKV infection was also highest in Sudan, with anti-CHIKV antibody prevalence by HI or ELISA ranging from 1.8% to 30%. In Yemen, 0% of 222 cases of undifferentiated AFI were positive for CHIKV in the eastern coastal city of Al-Mukalla in June 2010 [35]. Four months later, however, an outbreak of CHIKV was detected in the western coastal governorate of Al-Hudaydah, Yemen, and two studies demonstrated 9.8% and 28% ELISA IgM seroprevalence among subjects with undifferentiated AFI [10, 36].

**Table 2:** Human prevalence studies for *Chikungunya virus* in the Middle East and North Africa (n = 29).

Country, Ref.	Year(s) of study*	City or Governorate	Setting; population (age range, years)	Sampling	Assay type^†^	Assay make	Sample size	Prevalence	Additional testing & Comments	
**Djibouti** (n = 4)										
Salah [27]	1987	Djibouti City	Military; healthy soldiers	Conv.	IIFT	In-house	50	**0%**	no additional testing performed	
		Randa	Rural community; general pop.	Conv.	IIFT	In-house	69	**0%**	no additional testing performed	
		Djibouti City	Hospital; AFI patients	Conv.	IIFT	In-house	41	**2.4%**	The single IIFT+ subject was a native of Ethiopia and was cross-reactive with SINV	
Andayi [31]	2010–11	Djibouti City	Household survey; general pop.(<1–100)	SRS	ELISA	In-house	914	**2.6%**	95.9% (23/24) ELISA+ were VNT+	
**Egypt** (n = 2)										
Darwish [41]	1974*	Multiple	n/s; general pop. (<1–70)	n/s	HI	In-house	231	**0%**	0% were SFV+; 16.8% were SINV+	
Darwish [37]	1985	Cairo	Hospital; AFI patients (>10)	Conv.	HI	In-house	55	**5.5%**	0% (0/55) were convalescent +; 0% were SINV+	
**Iran** (n = 2)										
Saidi [28]	1970	Multiple	n/s	n/s	HI	In-house	394	**22.1%**	no additional testing performed	
Saidi [42]	1970–71	Caspian region	Community; children (1–6)	Conv.	HI	In-house	100	**0%**	0% were SINV+	
**Iraq** (n = 1)									
Barakat [43]	2012–13	Nasiriyah	Community; healthy medical staff, blood donors, students, non-AFI patients (10–82)	n/s	IIFT	In-house	399	**0.5%**	2 of 4 ambiguous CHIKV+ samples were VNT+; 2% (8/399) of CHIKV+ were SINV+; 6/8 SINV+ were VNT+; all SINV+ and CHIKV+ samples were negative for SFV by VNT
**Kuwait** (n = 2)										
Ibrahim [29]	1966–68	Multiple	Multiple; blood donors, non-AFI patients, children (1–60)	Conv.	HI	In-house	627	**1.4%**	78% (7/9) CHIKV+ samples were cross-reactive to SINV and SFV; 4.5% (28/627) were SINV+; 2.6% (16/627) were SFV+	
Al-Nakib [30]	1979–82	Jabriya	Hospital; non-AFI patients (0–60+)	SRS	HI	In-house	502	**0.4%**	100% (2/2) CHIKV+ samples were cross-reactive with SINV	
**Pakistan** (n = 2)										
Darwish [26]	1983*	Karachi	Hospital; patients	Conv.	CF	In-house	43	**2.3%**	2.3% were SINV+; possible CHIKV cross-reaction with SINV	
Afzal [44]	2011	Lahore	Hospital; AFI patients (<12)	Conv	ELISA	n/s	75	**4%**	no additional testing performed
**Somalia** (n = 1)										
Botros [38]	1987	Hargeysa	Refugee camp; AFI patients	Conv.	HI	In-house	28	**0.0%**	0% (0/10) convalescent samples tested were HI+; 0% (0/28) were HI+ for SINV	
**Sudan** (n = 11)										
Salim [32]	1973*	Sennar	Community and clinical setting; general pop. and non-AFI patients (<1–40+)	Conv.	VNT	In-house	62	**12.9%**	23% (11/48) were also VNT+ for ONNV	
Omer [33]	1976	Gezira State	Rural community; general pop. (5–40+)	Conv.	HI	In-house	109	**24.8%**	0.9% (1/109) were also HI+ to SINV; 8.2% (9/109) were VNT+ for CHIKV	
Woodruff [45]	1986	Juba	Hospital; patients with history of fever within past 6 months and AFI patients (1–85)	Conv.	HI	In-house	130	**23.1%**	3.1% (4/130) were HI+ for SINV; 2.3% (3/130) were HI+ for SFV; no observed cross-reaction between CHIKV and SINV or SFV; 1 observed cross-reaction between SINV and SFV	
McCarthy [46]	1988	Khartoum	Clinical setting; non-AFI patients	Conv.	ELISA	In-house	100	**11%**	1/100 (1%) were IgM+	
		Khartoum	Clinical setting; AFI patients (1–89)	Conv.	ELISA	In-house	196	**10%**	1/200 (0.5%) were IgM+	
Watts [47]	1989	Northern state	Clinical setting; AFI patients (11–70)	Conv.	ELISA	In-house	185	**12.0%**	no additional testing performed	
Farnon [14]	2005	Kortalla	Community; general pop. (0–44+)	SSCS	ELISA	In-house	87	**43%**	1% (1/87) was CHIKV IgM+; 7.9% (3/38) CHIKV+ samples were SINV+	
Gould [48]	2005	South Kordofan	Clinical setting; suspected YF patients (n = 3), severe illness (n = 8), AFI patients (n = 7), healthy (n = 16)	Conv.	ELISA IgM	In-house	34	**23.5%**	no additional testing performed	
Adam [34]	2012–13	Eastern and Central Sudan	Clinical setting; AFI patients (<15–45+))	Conv.	ELISA	Euroimmun	379	**1.8%**	All ELISA+ were also IFA+ and VNT+
Baudin [49]	2011–12	Port Sudan	Hospital; pregnant women with fever	Conv.	qRT-PCR	In-house	130	**30%**	8 of 39 CHIKV+ patients were also positive for *Rfit Valley Fever virus* by PCR or IgM ELISA
Enkhtsetseg [50]	2012–13	South Sudan	Military; military seroconversion study over ~6 month period	Conv.	HI	In-house	632	**0%**	no additional testing was performed
**Tunisia** (n = 1)										
Nabli [51]	1970*	Multiple	n/s; children	Conv.	HI	In-house	100	**0%**	0.2% (3/1406) were HI+ for SINV	
**Yemen** (n = 3)										
Madani [35]	2010	Hadramout	Clinical settings; suspected viral hemorrhagic fever (3–75)	Conv.	RT-PCR	In-house	222	**0%**	no additional testing performed	
Malik [10]	2010–11	Al-Hudaydah	Clinical setting; AFI patients (0–45+)	Conv.	ELISA IgM	In-house	136	**28%**	40% (54/136) were RT-qPCR+; 22% (30/136) were cell culture +	
Rezza [36]	2012	Al Hudaydah	Hospitals; AFI patients with ‘dengue-like’ illness (1–60)	Conv	ELISA IgM	NovaLisa	400	**9.8%**	2.8% (11/400) were RT-qPCR+; 9.4% (33/351 negative IgM/PCR) were IgG+	

* Indicates year of publication when year(s) of data collection not available in report.

^**†**^ All serologic assays were IgG unless otherwise stated.

Abbreviations: AFI, acute febrile illness patients; CF, complement fixation; Conv, convenience; ELISA, enzyme-linked immunosorbent assay; HI, hemagglutinin inhibition; IFA, indirect fluorescent antibody, IIFT, indirect immunofluorescence test; n/s, not specified; ONNV, *O’nyong-nyong virus*; pop., population; PCR, polymerase chain reaction; RT-qPCR, quantitative reverse transcription PCR; SFV, *Semliki Forest virus*; SINV, *Sindbis virus*; SRS, simple random sampling; SSCS, single stage cluster sampling; VNT, viral neutralization test

Assay Abbreviation: *NovaLisa* (Dietzenbach, Germany)

Convalescent sera results were reported in two AFI studies, neither of which was positive as indicated by a four-fold rise in antibody titers [37, 38]. One study reporting detection of CHIKV in mosquito pools was identified in our search, in which CHIKV was detected by RT-qPCR in 26.6% of 11 pools of 30 *Ae*. *aegypti* mosquitoes that were collected at an Eritrean refugee camp in Al Hudaydah, Yemen during the aforementioned CHIKV outbreak [39]. In this study, mosquitoes were collected by BG-sentinel^TM^ traps, Knock-down pyrethroid spray, and indoor aspirations from houses of recently reported cases, and larvae were inspected in all possible containers per house or inhabited premises. The total container index of the sampled sites was 53.8, Breteau index was 100, and house index was 57. *Ae*. *aegypti* adult female minimum and maximum infection rates were 20% and 72%, respectively. There were no published reports of single CHIKV isolations from mosquitoes or population-based human incidence identified in our search. However, during the 2012 CHIKV outbreak in southern Yemen, one study reported an overall CHIKV attack rate of 7.5 per 1,000 people, ranging from 5.3 among children 0–4 years of age to 12.2 among adults ≥ 45 years [40].

### Precision and risk of bias assessment results

The quality assessment for each prevalence study is found in Table 3. Overall, 66% (19/29) of studies contained high precision as defined by a sample size of ≥100 subjects. For the risk of bias assessment, response rates were ≥80% in 14% (4/29) of studies, <80% in 3% (1/29) of studies, and not reported in 83% (24/29) of studies. Among general seroprevalence studies, some form of probability sampling (i.e. low ROB) was reported in 31% (5/16) of studies, non-probability sampling (e.g. high ROB) in 31% (5/16) of studies, and unclear sampling methods in 38% (6/16) of studies.

**Table 3:** Precision and risk of bias assessment for *Chikungunya virus* prevalence measures in the Middle East and North Africa.

Country, Ref.	Year(s) of study	Population	Risk of Bias Assessment		Precision
			Sampling*	Response rate	
**Djibouti**					
Salah [27]	1987	Healthy soldiers	High ROB	Unclear ROB	Low
		General population	High ROB	Unclear ROB	Low
	1987	AFI patients	n/a	Unclear ROB	Low
Andayi [31]	2010–11	General population	Low ROB	High ROB	High
**Egypt**					
Darwish [41]	1974	General population	Unclear ROB	Unclear ROB	High
Darwish [37]	1985	AFI patients	n/a	Unclear ROB	Low
**Iran**					
Saidi [28]	1970	n/s	Unclear ROB	Unclear ROB	High
Saidi [42]	1970–71	Children	Low ROB	Unclear ROB	High
**Iraq**					
Barakat [43]	2012–13	General population, blood donors, non-AFI patients	Unclear ROB	Unclear ROB	High
**Kuwait**					
Ibrahim [29]	1966–68	Blood donors, non-AFI patients, children	Low ROB	Unclear ROB	High
Al-Nakib [30]	1979–82	non-AFI patients	Low ROB	Unclear ROB	High
**Pakistan**					
Darwish [26]	1983	Hospital patients	Unclear ROB	Unclear ROB	Low
Afzal [44]	2011	AFI patients	n/a	Unclear ROB	Low
**Somalia**					
Botros [38]	1987	AFI patients	n/a	Unclear ROB	Low
**Sudan**					
Salim [32]	1973*	General population and non-AFI patients	Unclear ROB	Unclear ROB	Low
Omer [33]	1976	General population	High ROB	Unclear ROB	High
Woodruff [45]	1986	AFI patients	n/a	Unclear ROB	High
McCarthy [46]	1988	Non-AFI patients	High ROB	Unclear ROB	High
		AFI patients	n/a	Low ROB	High
Watts [47]	1989	AFI patients	n/a	Unclear ROB	High
Farnon [14]	2005	General population	Low ROB	Unclear ROB	Low
Gould [48]	2005	AFI patients	n/a	Low ROB	Low
Adam [34]	2013–13	AFI patients	n/a	Unclear ROB	High
Baudin [49]	2011–12	Pregnant women with AFI	n/a	Unclear ROB	High
Enkhtsetseg [50]	2012–13	Military	High ROB	Unclear ROB	High
**Tunisia**					
Nabil [51]	1970	Children	Unclear ROB	Unclear ROB	High
**Yemen**					
Madani [35]	2010	Suspected viral hemorrhagic fever	n/a	Low ROB	High
Malik [10]	2010–11	AFI patients	n/a	Low ROB	High
Rezza [36]	2012	Patients with dengue-like illness	n/a	Unclear ROB	High

* Since the populations of acute febrile illness or suspected arbovirus infection are defined as populations presenting to a health facility with acute infection, no population-based sampling is needed to capture these populations and they are denoted ‘n/a’ in the sampling column.

Abbreviation: AFI, acute febrile illness

A total of four CHIKV outbreaks, three case reports/series, and one report of CHIKV in returned travelers, were identified through the search databases (Table 4) and were mapped along with the geographic distribution of prevalence studies (Fig 2). Outbreaks were reported from Djibouti, Sudan, Pakistan, and Yemen. In most cases, the vector was suspected to be *Ae*. *aegypti* given its known occurrence in the affected countries, but was only confirmed as such in the 2011–2012 outbreak in Yemen [10].

**Table 4:** Summary of reported outbreaks, case series, case reports, and cases in travelers for *Chikungunya virus* in the Middle East and North Africa.

Country, Year	City or Governorate	Description	Ref.
**Djibouti**			
2011	Djibouti City	Chikungunya outbreak reported was concurrent with 2011 outbreak in Yemen; *Aedes aegypti* was the suspected vector.	[31]
**Pakistan**			
2016–17	Karachi	A total of 2,267 reported cases during an outbreak from December 2016 to May 2017. *Aedes aegypti* was the suspected vector.	[52]
**Saudi Arabia**			
2011	Jeddah	First autochthonous case of chikungunya detected by qRT-PCR in Saudi Arabia; unconfirmed vector.	[53]
**Somalia**			
2016	Mogadishu	Two travelers returning to Italy from Mogadishu, Somalia. For both patients, testing was positive by CHIKV IFA IgG and IgM (Euroimmun), Anti-CHIKV IgM ELISA (Euroimmun), and PRNT.	[54]
2016	Mogadishu	11 cases were confirmed by RT-PCR, representing the first reports of human CHIKV infection by Somalia; unconfirmed vector.	[55]
**Sudan**			
2005	South Kordofan	Concurrent chikungunya transmission detected during yellow fever outbreak; *Aedes aegypti* was the suspected vector.	[48]
2014	Not specified	16 cases were reportd from Sudan in 2014; unconfirmed vector	[55]
2015	Darfur	4 laboratory-confirmed cases were reported from Sudan in 2015; unconfirmed vector	[56]
**Yemen**			
2011–12	Al Hudaydah, Lahj	Over 15,000 suspected cases during an outbreak; *Aedes aegypti* was the proven vector.	[10]

## Discussion

Our review examines the epidemiology of urban CHIKV in the MENA region by summarizing published human prevalence and incidence studies, outbreaks, reported cases, and reports of CHIKV detected in *Aedes* mosquitoes. Serologic and outbreak evidence of CHIKV transmission has been identified in the countries surrounding the Red Sea (Djibouti, Egypt, Saudi Arabia, Somalia, Sudan, and Yemen) and Pakistan. This distribution overlaps the known distribution of DENV in the MENA region [15]. No outbreaks, cases of autochthonous transmission, or human seroprevalence studies were reported from 13 of 23 countries in the MENA region, while a single seroprevalence studies in Tunisia [51] did not identify anti-CHIKV antibodies in the populations tested. Given increasing reports of *Ae*. *aegypti* and *Ae*. *albopictus* occurrence and DENV transmission in the MENA over recent years, the possibility of unrecognized CHIKV transmission or cross-border spread of CHIKV to these countries merits consideration.

As the bulk of epidemiologic evidence for CHIKV is serologic, the genetic diversity of CHIKV circulating in the MENA is largely unknown. To date, the only published CHIKV strain from the MENA comes from the 2011 Yemen outbreak [10]. This virus was found to be similar to the East-Central-South African (ECSA) Indian Ocean Lineage that was responsible for the 2004–2006 Indian Ocean epidemic [11, 12] and subsequent urban outbreaks in Southeast Asia, Italy, and France [57]. Still, CHIKV lineages may be diverse within North Africa and distinct from other regions in Africa. Distinct enzootic / epidemic ECSA lineages may be circulating in Sudan, for example, given the repeated detection of anti-CHIKV antibodies in Sudanese populations since the 1970s [32, 33], Sudan’s proximity to East Africa (where CHIKV has long been endemic), and the sylvatic habitats in Sudan’s Nuba Mountain region where several yellow fever outbreaks may have emerged from in the past [14, 32, 58–60]. Contiguous spread of CHIKV from India to Pakistan is similarly plausible, as is import-related outbreaks from more remote regions, such as from South Asia to the Arabian Peninsula or from Mediterranean Europe to North Africa.

A variety of ecologic and social risk factors may be driving the spread of *Aedes* in the MENA, which we describe in detail in our recent publication on DENV in the MENA [15]. These factors include increasing urbanization [61], use of open water storage that encourages *Ae*. *aegypti* breeding [39, 62], armed conflict and poverty [63, 64], and extensive inter- and intra-regional trade and migration [12, 65]. The 2011 CHIKV outbreak in Yemen illustrates several vulnerabilities to *Aedes*-borne arboviruses potentially faced by the MENA [10]. Heavy travel and commerce through the port city of Al Hudaydah was thought to have led to CHIKV introduction, possibly from North African port cities along the Red Sea coast. A CHIKV outbreak was reported in Djibouti City [31] the same year of the Yemen outbreak, and the following year, autochthonous transmission of CHIKV was noted in Saudi Arabia [53]. Similar transmission patterns have been cited as risk for DENV infection spread in the Red Sea sub-region [10, 12, 27, 66, 67]. High rainfall in Al Hudaydah months prior to the outbreak may have also amplified the *Aedes* population to a level capable of sustaining an outbreak. The outbreak was not detected until nearly 16 weeks after its suspected onset, in part attributable to a lack of surveillance and case detection, and initial misrecognition of the disease as DENV in a known dengue-endemic area [10]. Prompter initiation of vector control measures may have forestalled the amplification of the outbreak over the following months.

Serologic cross-reactions for alphaviruses like CHIKV are well documented and represent an important limitation of epidemiologic studies which rely on serology in the absence of confirmatory neutralization tests. Serologic evidence of CHIKV in the absence of neutralization tests must be interpreted with caution, as such results may represent cross-reactions between CHIKV and another alphavirus (e.g. SINV, ONNV, or SFV), co-cicrulation of both viruses, cross neutralization between both viriuses, or the presence of another antigenically-related virus that induces cross-neutralizing antibodies to both alphaviruses [68]. These alphaviruses may have similar clinical presentations and geographic distributions in the MENA region, while the increase in international travel, intensification of trade, and the spread of potential vectors as a result of climate change may further drive overlapping endemicity [69]. In our review, serologic cross-reactions were reported in 9 of 14 seroprevalence studies, most commonly due to SINV, another Old World alphavirus identified in the MENA whose epidemiologic impact is also poorly understood [70]. Like *West Nile Virus*, SINV may be broadly distributed in the MENA given its enzoonotic transmission cycle involving migratory avian hosts and ubiquitous *Culex* mosquitoes [71–73]. In a study by Ibrahim et al. in Kuwait, 78% (7/9) CHIKV positive samples were cross-reactive to SINV and SFV by HI testing, while cross-reactivity between SINV and SFV occurred in an additional 9 of 21 SINV-reactive sera [39]. In a second study from Kuwait, all CHIKV positive samples were cross-reactive to SINV [30]. This evidence does not confirm CHIKV circulation in Kuwait. Antibodies against ONNV, another Old World alphavirus whose distribution is likely underreported, were only sought (and confirmed by VNT) in one study from Sudan conducted in 1973 [32]. Yet although the distribution of ONNV in the MENA region requires further research, it is unlikely that the serologic studies reporting CHIKV are primarily detecting ONNV. A one-way antigenic cross-reactivity has been described between CHIKV and ONNV, in which antibodies against CHIKV neutralize ONNV but anti-ONNV antibodies do not recognize CHIKV [74, 75]. Further assurance of the detection of CHIKV antibodies is found in the study by Andayi et al. in Djibouti, in which a non-inactivated viral antigen was utilized for ELISA testing (allowing for selection of more specific anti-CHIKV antibodies) and results were confirmed by seroneutralization [31].

CHIKV may continue to spread in the MENA region as it has in other regions. Surveillance programs and clinicians should maintain alertness for CHIKV, particularly in dengue-endemic areas, given the overlapping clinical presentation and geographic distribution of both pathogens. Addressing this challenge will require multiplexed serologic and molecular diagnostics able to simultaneously detect and discriminate between CHIKV and other pathogens with overlapping clinical manifestations, such as DENV, malaria, ONNV, *Yellow fever virus*, and *Zika virus* [2, 10, 13, 14, 48, 76–80]. Such diagnostics could also circumvent the issue of cross-reactivity and the frequent unavailability of convalescent serology. However, molecular diagnostics are limited by the presence of viral RNA or antigen for a limited duration during acute infection. Studies to understand the distribution of *Aedes* and infection rates in *Aedes* in the MENA are also important, as they can inform our understanding of transmission dynamics which is useful for vector control strategies and predicting future transmission risk [71–73]. Our review identified only one published study of CHIKV isolated from *Aedes* in the region [39], depite the published occurrence of *Ae*. *aegypti* and *Ae*. *albopictus* in 11 and 7 MENA countries, respectively [15].

Our study was limited by its use of select databases of peer-reviewed literature with the possible exclusion of some grey literature which may have provided additional data (the African Index Medicus and Index Medicus for the Eastern Mediterranean Region databases do index grey literature). Reports from public health laboratories in each country may also have provided additional data, though we chose to focus our review on published literature that would be available to the broader academic community in the region. In addition, although we did not impose any publication year restrictions in our search, Embase and the regional databases began indexing articles after 1980, so earlier publications may have been overlooked during our search. PubMed began indexing articles prior to the discovery of CHIKV in 1952–53, however. The urban cycle focus of CHIKV epidemiology must also be underscored in our manuscript, as we did not include seroprevalence studies in animals (i.e. sylvatic cycle) in our review criteria. Still, only one seroprevalence study in animals was identified in our search. It is likely that our study generally reflects the available seroprevalence literature concerning CHIKV in the MENA, as we identified no mention of sylvatic cycle CHIKV in the MENA over the course of our review. CHIKV may primarily exist in a human-peridomestic mosquito cycle in the MENA, as it existis in Asia [6], though sylvatic cycle CHIKV is known to exist elsewhere in Africa [81]. Non-publication of studies with small or zero effect size may also have biased the distribution of studies identified in our review. The absence of confirmatory neutralization tests in many studies is an additional limitation, along with the lack of convalescent serum tests to demonstrate antibody titer kinetics in acute infections, both of which would reduce the likelihood of cross-reaction. Indeed, serologic cross-reactions may have overestimated CHIKV seroprevalence in multiple studies. Finally, given the small number of studies and sparse distribution and content of available studies, we did not perform a meta-analysis or explore bias in overall outcome measures through a funnel plot or Egger test nor did we exclude studies based on the risk of bias assessment. This resulted in the inclusion of studies of varying methodological rigor and scientific quality.

### Conclusions

In the MENA region, published reports suggest endemic circulation of CHIKV in the Red Sea Region and Pakistan, similar to the known geographic distribution of DENV. Published epidemiologic studies are lacking in many sub-regions, including some *Aedes* and DENV endemic areas, further suggesting underrecognition of CHIKV in the MENA. These findings articulate the need for further research to understand the epidemiology of CHIKV in the MENA.

## Supporting information

S1 Fig Data sources and search criteria used for the systematic review of *Chikungunya virus* in the Middle East and North Africa. (PDF)

S1 Table Preferred Reporting Items for systematic reviews and meta-analyses (PRISMA) checklist. (PDF)
