# Supplementary material for: Urban Chikungunya in the Middle East and North Africa: A systematic review
Source: PLoS Negl Trop Dis. 2017 Jun 26;11(6):e0005707. doi: 10.1371/journal.pntd.0005707 (PMC5501693; doi:10.1371/journal.pntd.0005707)
Supplement: S1 Fig — (PDF) [file pntd.0005707.s001.pdf]

Chikungunya Search Criteria
**PubMed** (Last searched May 2, 2017)
("Chikungunya"[text] OR "Chikungunya Virus"[mesh] OR “CHIK”[text] OR Alphavir*[text] OR “Arbovirus, Group A”[Mesh]) **AND** ("Middle East"[Mesh] OR "Islam"[Mesh] OR "Arabs"[Mesh] OR "Arab World"[Mesh] OR "Africa, Northern"[Mesh] OR "Sudan"[Mesh] OR "Somalia"[Mesh] OR "Djibouti"[Mesh] OR "Pakistan"[Mesh] OR "Middle East"[Text] OR "Middle-East"[Text] OR "North Africa"[Text] OR "North-Africa"[Text] OR "EMRO"[Text] OR "Eastern Mediterranean"[Text] OR “Arab”[Text] OR “Arabs”[Text] OR “Arab World”[Text] OR "Islam"[Text] OR "Afghanistan"[Text] OR "Algeria"[Text] OR "Bahrain"[Text] OR "Djibouti"[Text] OR "Egypt"[Text] OR "Jordan"[Text] OR "Kuwait"[Text] OR "Lebanon"[Text] OR "Libya"[Text] OR "Iran"[Text] OR "Iraq"[Text] OR "Morocco"[Text] OR "Oman"[Text] OR "Pakistan"[Text] OR "Qatar"[Text] OR "Saudi Arabia"[Text] OR "Somalia"[Text] OR "Sudan"[Text] OR "Syria"[Text] OR "Tunisia"[Text] OR “Turkey”[text] OR "United Arab Emirates"[Text] OR "Dubai"[Text] OR "Abu Dhabi"[Text] OR "Abu-Dhabi"[Text] OR “Sharjah”[Text] OR "West Bank"[Text] OR "Ghaza"[Text] OR "Palestine"[Text] OR "Yemen"[Text])
**Embase** (Last searched May 2, 2017)
(exp Chikungunya/ OR exp Chikungunya virus/ OR Chikungunya.mp OR CHIK.mp OR Alphavir*.mp or group A arbovirus.mp) **AND** (exp Middle East/ or exp North Africa/ or exp Arab/ or exp Afghanistan/ or exp Djibouti/ or exp Pakistan/ or exp Somalia/ or exp Sudan/ or Middle East.mp. or North Africa.mp. or EMRO.mp. or Eastern Mediterranean.mp. or Arab.mp. or Arabs.mp. or Arab World.mp. or Islam.mp. or Afghanistan.mp. or Algeria.mp. or Bahrain.mp. or Djibouti.mp. or Egypt.mp. or Jordan.mp. or Kuwait.mp. or Lebanon.mp. or Libya.mp. or Iran.mp. or Iraq.mp. or Morocco.mp. or Oman.mp. or Pakistan.mp. or Qatar.mp. or Saudi Arabia.mp. or Somalia.mp. or Sudan.mp. or Syria.mp. or Tunisia.mp. or Turkey.mp or United Arab Emirates.mp. or Dubai.mp. or Abu Dhabi.mp. or Sharjah.mp. or West Bank.mp. or Ghaza.mp. or Palestine.mp. or Yemen.mp.)
**Regional Databases**
**Index Medicus for the Eastern Mediterranean Region, African Index Medicus, and ProMED-MENA** (Last searched May 13, 2017)
a search was conducted using only the term ‘chikungunya’
